# Endoscopic Management of Nonlifting Colon Polyps

**DOI:** 10.1155/2013/412936

**Published:** 2013-05-14

**Authors:** Shai Friedland, Andrew Shelton, Shivangi Kothari, Rajan Kochar, Ann Chen, Subhas Banerjee

**Affiliations:** ^1^Department of Medicine, Stanford University, Stanford, CA, USA; ^2^Gastroenterology Section, VA Palo Alto HCS, Palo Alto, CA, USA; ^3^Department of Surgery, Stanford University, Stanford, CA, USA

## Abstract

*Background and Study Aims*. The nonlifting polyp sign of invasive colon cancer is considered highly sensitive and specific for cancer extending beyond the mid-submucosa. However, prior interventions can cause adenomas to become nonlifting due to fibrosis. It is unclear whether nonlifting adenomas can be successfully treated endoscopically. The aim of this study was to evaluate outcomes in a referral practice incorporating a standardized protocol of attempted endoscopic resection of nonlifting lesions previously treated by biopsy, polypectomy, surgery, or tattoo placement. *Patients and Methods*. Retrospective review of patients undergoing colonoscopy by one endoscopist at two hospitals found to have nonlifting lesions from prior interventions. Lesions with biopsy proven invasive cancer or definite endoscopic features of invasive cancer were excluded. Lesions ≥ 8 mm were routinely injected with saline prior to attempted endoscopic resection. Polypectomy was performed using a stiff snare, followed by argon plasma coagulation (APC) if necessary. *Results*. 26 patients each had a single nonlifting lesion with a history of prior intervention. Endoscopic resection was completed in 25 (96%). 22 required snare resection and APC. 1 patient had invasive cancer and was referred for surgery. The recurrence rate on follow-up colonoscopy was 26%. All of the recurrences were successfully treated endoscopically. There was 1 postprocedure bleed (4%), no perforations, and no other complications. *Conclusions*. The majority of adenomas that are nonlifting after prior interventions can be treated successfully and safely by a combination of piecemeal polypectomy and ablation. Although recurrence rates are high at 26%, these too can be successfully treated endoscopically.

## 1. Introduction

Submucosal injection of saline or other substances is commonly used during endoscopic resection of colon polyps as it may reduce the risk of inadvertent perforation or thermal injury to the muscularis propria [1, 2]. While most lesions separate from the muscularis propria and lift in response to submucosal injection, others do not and are considered nonlifting. The nonlifting sign of invasive colon cancer was first described in 1994 by Uno and Munakata [3]. In their original article, they reported that submucosal injection of saline with methylene blue beneath invasive cancers did not result in lifting of the lesions, while injection beneath adenomas lifted them. Subsequent reports further refined these observations [4–6]. In the absence of prior endoscopic interventions, adenomas, mucosal carcinomas, and early cancers with superficial submucosal invasion (sm1, sm2) generally lift, whereas cancers extending to the deep submucosa (sm3) or beyond do not. However, prior interventions such as partial polypectomy, submucosal injection, tattoo, and biopsy can lead to fibrosis and result in nonlifting of lesions that would otherwise lift. Han et al. reported that it takes approximately 3 weeks for fibrosis to develop after biopsy, so that if repeat colonoscopy is performed within 3 weeks lesions that were previously biopsied can be lifted [5].

The nonlifting sign is widely considered to be useful for two reasons. The first is that nonlifting cancers with sm3 or deeper invasion carry a substantial risk of lymph node metastasis, so surgical management is appropriate, and endoscopic resection will generally not obviate the need for surgery [7–9]. The second is that nonlifting lesions are considered challenging and perhaps risky to resect endoscopically. Submucosal fibrosis can make it difficult to grasp these lesions with a snare, and there is a risk of cutting through the adherent muscularis propria and perforating the colon if the lesion is snared. For these reasons, endoscopists have been cautioned repeatedly to avoid resecting nonlifting lesions, to avoid biopsy of lesions that may be referred to expert centers for endoscopic resection and to expedite repeat colonoscopy for resection of those lesions that have been biopsied, so that resection can be performed before the 3 weeks period during which fibrosis develops [10–12]. However, there is very little data regarding the efficacy and risk of endoscopic treatment of nonlifting lesions.

We have observed that in our western practice many colorectal lesions referred for endoscopic resection have been disturbed by interventions such as prior biopsy, partial polypectomy, and submucosal injection of India Ink tattoo. Furthermore, in nearly all cases more than 3 weeks have elapsed between the initial colonoscopy and the resection procedure. In this report we describe our experience with nonlifting colorectal lesions.

## 2. Methods

After obtaining institutional review board approval for the study, a retrospective chart review was performed of all colonoscopies performed by a single endoscopist (SF) at Stanford University Hospital and the Veterans Affairs Palo Alto Medical Center between January 2011 and May 2012. Complete electronic databases of all procedures performed are available at both hospitals. The databases were used to extract and manually review the procedure reports and photographs in all colonoscopy and sigmoidoscopy procedures performed by the endoscopist during the study period. The endoscopist has extensive experience in endoscopic resection and has performed over 1000 endoscopic mucosal resection (EMR) procedures in the past 10 years. During this period, a standardized resection procedure was used in all cases. Lesions with definite endoscopic features of advanced cancer—such as ulcerated and excavated tumors—were biopsied and referred for surgery. All remaining lesions 8 mm or greater were routinely injected with saline stained blue with trace indigo carmine prior to endoscopic resection. Additional injection with 1 : 100,000 epinephrine was used during piecemeal endoscopic resection if bleeding was encountered with initial polypectomy. Photographs were routinely taken after submucosal injection to demonstrate lifting or nonlifting, and any instances of nonlifting were documented in the procedure report. All lesions encountered during this time period with nonlifting of part or all of the lesion and a history of prior intervention were included in this study.

Polypectomy of nonlifting lesions was performed using a stiff snare (Traxtion, US Endoscopy, Mentor, OH, USA; SD-230 or SD-210, Olympus America, Center Valley, PA, USA). Cautery settings were not standardized. Areas of residual lesion that could not be grasped with a standard stiff snare were removed if possible using a miniature cold snare (Exacto, US Endoscopy, Mentor, OH, USA). Areas of residual lesion that could still not be removed were treated with argon plasma coagulation (APC) at a setting of 40–60 Watts (ERBE USA, Marietta, GA, USA). All patients were followed up by clinic visit or telephone at least 10 days after the procedure to assess for bleeding, perforation, or other complications.

## 3. Results

During the study period, the endoscopist performed a total of 767 colonoscopies and sigmoidoscopies. A total of 235 polyps 8 mm or larger were found on 192 of these procedures. Submucosal injection was performed on 199 of these lesions (85%). Submucosal injection was not performed on 36 (15%) lesions for various reasons: 5 were removed by cold snare, 6 pedunculated lesions were snared without cautery to facilitate placement of the snare on the stalk, 16 were in patients enrolled in a prospective study of an alternative resection technique, 5 were not removed due to advanced patient age and comorbidities, and 4 were felt to be endoscopically unresectable and therefore not injected. 30 of the 199 lesions that underwent submucosal injection did not lift (15%). Of these, 4 were excluded from the study because they had not undergone prior intervention; 26 lesions in 26 patients were therefore found to have nonlifting lesions with a history of prior intervention and were included in the study. In 2 of the 4 excluded nonlifting patients without prior intervention, diagnostic EMR demonstrated invasive cancer, so they were referred for definitive surgery. One excluded patient had successful piecemeal EMR of a villous adenoma with high-grade dysplasia. He had a 3 mm residual adenoma without high-grade dysplasia on 3-month followup and no residual at 12- and 18-month follow-up colonoscopies. The fourth excluded patient underwent piecemeal EMR with histology demonstrating tubular adenoma without high-grade dysplasia. He refused further followup. 

**Figure 1:**
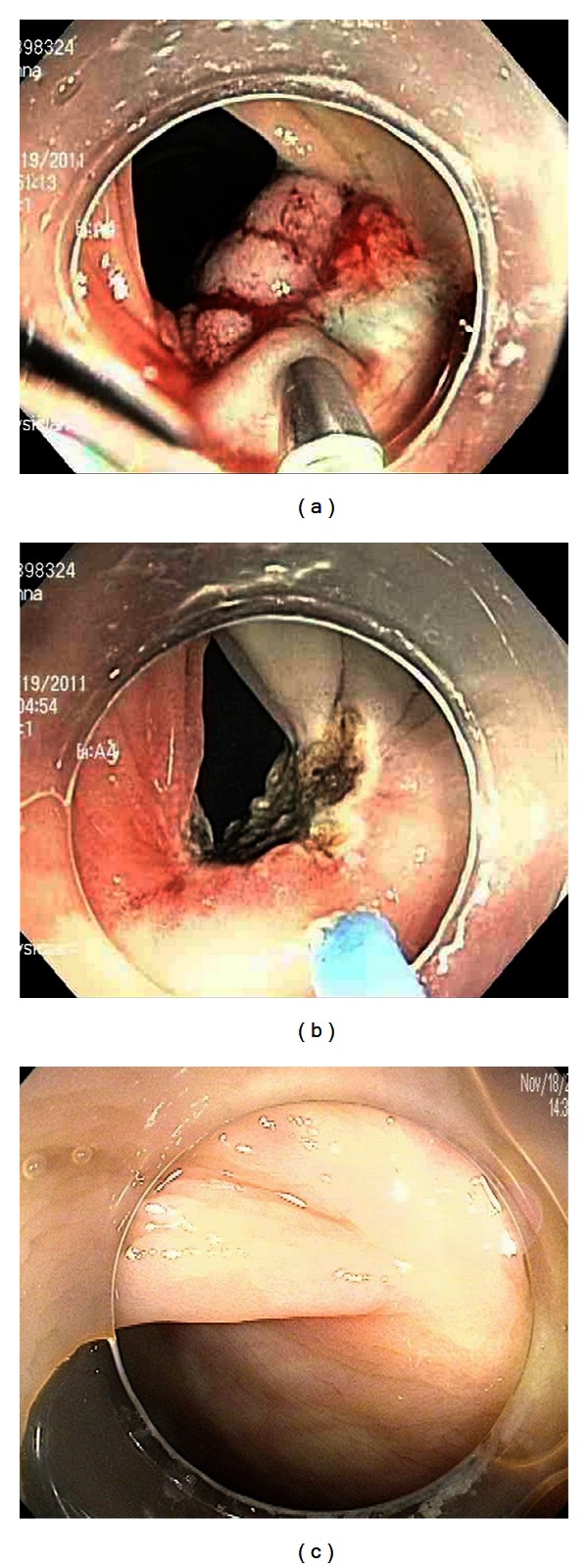
(a) Sigmoid villous adenoma. Piecemeal polypectomy was attempted twice at an outside facility prior to referral. The lesion demonstrates nonlifting with indigo carmine stained saline raising only the normal mucosa adjacent to the lesion. (b) Appearance of the site immediately after polypectomy and argon plasma coagulation. (c) There was no recurrence on follow-up procedures.

Table 1 shows the demographics of the 26 patients and the characteristics of the 26 lesions. 17 were male (65%). The median age was 68 years. 21 of the patients were referred specifically for resection of the colon lesion; the other 5 patients were referred for surveillance after prior polypectomy or surgery. The lesions ranged in size from 10 to 50 mm, with a median of 20 mm. 12 of the lesions (46%) were completely nonlifting, while 11 (42%) had nonlifting of one side, and 3 (12%) had nonlifting of the central area of the lesion. Figure 1 shows a representative lesion, a villous adenoma that had undergone two prior attempts at piecemeal polypectomy that were incomplete. 

In 17 (65%) of the lesions, polypectomy had been attempted by the referring physician; in these cases only part of the lesion was removed, and the patient was referred for another attempt at completing endoscopic resection. 4 (15%) of the lesions were noted to have fibrosis at an area where the lesion was tattooed; in these cases the tattoo extended into the area of the lesion itself. Three patients (12%) had prior biopsies of the lesion, but no other potential iatrogenic cause for nonlifting. One patient with familial polyposis had a lesion at a surgical anastomosis from a prior subtotal colectomy. One patient had a lesion that extended inside a diverticulum. 

In 24 cases (92%), the endoscopist at our referral center considered the entire lesion to be sufficiently treated either by polypectomy alone (3 cases) or in combination with argon plasma ablation (21 cases). In one patient (4%) with a 50 mm cecal lesion partially extending into the terminal ileum, endoscopic resection was abandoned midway through the resection as the risk of perforation was felt to be too high. Histology demonstrated a villous adenoma without high-grade dysplasia. The patient had extensive comorbidities and chose not to undergo surgery. One patient (4%) with a 50 mm serrated adenoma containing multifocal cancer on piecemeal polypectomy was referred to surgery and was found to have a 2 mm focus of residual cancer at the resection site (T1N0). 

There was 1 complication (4%), a postpolypectomy bleed that resolved with conservative treatment. There were no perforations. 24 of the 26 patients were recommended to have follow-up surveillance; the patient with T1N0 cancer that was treated surgically and the patient with the endoscopically unresectable villous adenoma were not rescheduled due to advanced age and comorbidities. 19 of the 24 patients (79%) complied with the recommendation and underwent follow-up colonoscopy. 5 of the 19 (26%) had residual or recurrent adenoma at the resection site. 4 of these patients had diminutive adenomas measuring <5 mm. One patient had a 13 mm area of residual adenoma on followup after piecemeal resection of a 40 mm adenoma. All of the recurrences were treated endoscopically without complications. Two of the five patients have undergone a second followup, and in both cases there was no residual.

## 4. Discussion

The nonlifting sign of invasive colon cancer has been described extensively in multiple reports from centers in Japan and Korea [3–5]. It is widely established that it is both highly sensitive and highly specific for cancer extending into the deep submucosa or beyond in these expert centers when there has been no prior manipulation of the lesion. However, nonlifting can also be caused by biopsy, partial polypectomy, or tattooing of the lesion itself. At our center, lesions referred for endoscopic resection have often been disturbed by these types of interventions. Effective endoscopic treatment is highly desirable if there is a reasonable probability that the lesions are precancerous. In our series, only 1 of 26 patients had invasive cancer, justifying an aggressive endoscopic approach if safety and efficacy can be demonstrated. Overall, 24 of 26 patients (92%) were successfully treated by endoscopy, with only 1 postpolypectomy bleed (4%) and no perforations. Our experience therefore demonstrates that most of these nonlifting lesions that have been referred after prior manipulation can be treated relatively safely despite the lack of lifting.

Even when endoscopic treatment is not definitive, it can improve the accuracy of staging. In our series 1 patient (4%) was found to have invasive cancer and was appropriately referred for surgery. In 1 patient with a 50 mm cecal lesion involving the terminal ileum, endoscopic resection was not completed due to technical difficulty, but extensive sampling of the lesion demonstrated no invasive cancer or high-grade dysplasia, and based on this the patient chose to defer further treatment as he had severe medical comorbidities. However, endoscopists must be cautious regarding the possibility of potential understaging when lesions cannot be removed in one piece and are treated by piecemeal resection, or when ablation is used to treat areas that cannot be removed. Histology is limited in these cases and small foci of submucosal invasion or lymphovascular involvement can be missed; even if the endoluminal tumor is successfully treated lymph node metastasis can potentially occur and may not be detected in a timely manner. Endoscopic submucosal dissection (ESD) is a relatively new technique that is now well established for en bloc resection of early gastric cancer even in the setting of fibrosis [13]. ESD can also be used to successfully resect fibrotic colon lesions, but its use in this setting is technically challenging, risky, and time consuming [14–17]. While ESD reduces local recurrence rates compared to EMR [15, 16], until improvements in instrumentation and technique become available it is likely that EMR will still have a central role in the treatment of challenging colon lesions [18].

 Aggressive endoscopic treatment of nonlifting lesions in patients with prior interventions therefore offered significant clinical benefit with a low complication rate. The long-term outcome of these patients is less well established, but the available data is promising. After successful endoscopic resection demonstrating no invasive cancer in 24 patients, recurrent adenoma was found in 5 of the 19 (26%) patients that have undergone follow-up colonoscopy, but in all cases the adenoma was successfully resected endoscopically without complications. 2 of these 5 patients have subsequently undergone another follow-up colonoscopy, and none had evidence of recurrence. These results are comparable to those described following endoscopic mucosal resection of large sessile and flat adenomas. For example, in the large prospective multicenter Australian ACE study there was a recurrence rate of 13.5% for lesions up to 40 mm in diameter and a recurrence rate of 41% for lesions larger than 40 mm [19]. Data from the ACE study and other groups suggests that small areas of recurrence/residual adenoma are generally successfully treated with good results [15, 19, 20]. Future studies will need to address the long-term outcome of these patients more definitively, but as endoscopists throughout the world have developed expertise in piecemeal resection of large adenomas it is becoming increasingly clear that minute areas of recurrence are effectively addressed at follow-up procedures, so it is plausible that these patients will have good long-term outcomes.

Nearly all of the successful procedures in our series involved a combination of piecemeal polypectomy and argon plasma coagulation of remnants. Our experience and that of others suggests that it is likely that most of the lesions under 20 mm could have been resected more efficiently in 1 piece with a very low risk of residual if there was adequate lifting [21, 22]. Prior interventions causing fibrosis therefore increase the complexity of subsequent treatment, and we agree with the recommendation that it is best to avoid disturbing those lesions which will in any case be referred to a center specializing in endoscopic resection. However, our report highlights what we believe to be common practice in western colonoscopy programs: many endoscopists unsuccessfully attempt to resect lesions that are technically challenging, tattoos are too often placed too close to lesions, and biopsy of lesions is commonly performed before referral. We hope that future educational efforts will reduce these suboptimal practices and thereby facilitate endoscopic resection at future colonoscopies, but in the meantime our report suggests that nonlifting should be regarded as a manageable technical sequela of current practice style that in most cases can be addressed in expert centers without excessive morbidity for the patient. 

Limitations of the study include the limited number of patients, single endoscopist, retrospective design, and short-term followup. Despite these limitations, however, we suggest that when faced with a lesion that is nonlifting due to a prior intervention, it may be reasonable to proceed with endoscopic treatment if the patient is adequately informed of the risks, and the endoscopic team is sufficiently experienced.

## Figures and Tables

**Table 1:** Patient and lesion characteristics.

Patient age	Median 69 years, range 37–85
Sex	17 male (65%), 9 female (35%)
Lesion size	Median 20 mm, range 10–50 mm
Location	Right colon or transverse 17 (65%) Descending or sigmoid 8 (31%) Rectum 1 (4%)
Morphology	Sessile 18 (69%) Flat 8 (31%)
Prior therapy	Partial polypectomy 17 (65%) Tattoo under lesion 4 (15%) Biopsy only 3 (12%) Lesion at surgical anastomosis 1 (4%) Lesion involving diverticulum 1 (4%)
Nonlifting	Completely nonlifting 12 (46%) Nonlifting on one side 11 (42%) Nonlifting in center 3 (12%)
Pathology	Invasive cancer 1 (4%) High grade dysplasia 2 (8%) Adenoma 23 (88%)
Technical success	Piecemeal EMR with APC 19 (73%) One piece EMR with APC 3 (12%) One piece EMR without APC 3 (12%) EMR abandoned (technical inability) 1 (4%)
Complications	Bleeding 1 (4%) Perforation 0 (0%)

Recurrent adenoma 5 of 19 patients who had repeat colonoscopy (26%).
